# Mammals’ sperm microbiome: current knowledge, challenges, and perspectives on metagenomics of seminal samples

**DOI:** 10.3389/fmicb.2023.1167763

**Published:** 2023-04-17

**Authors:** María José Contreras, Kattia Núñez-Montero, Pablo Bruna, Ana Zárate, Felipe Pezo, Matías García, Karla Leal, Leticia Barrientos

**Affiliations:** ^1^Extreme Environments Biotechnology Lab, Center of Excellence in Translational Medicine, Universidad de La Frontera, Temuco, Chile; ^2^Facultad de Ciencias de la Salud, Instituto de Ciencias Biomédicas, Universidad Autónoma de Chile, Temuco, Chile; ^3^Escuela de Medicina Veterinaria, Facultad de Recursos Naturales y Medicina Veterinaria, Universidad Santo Tomás, Santiago, Chile; ^4^Scientific and Technological Bioresource Nucleus (BIOREN), Universidad de La Frontera, Temuco, Chile

**Keywords:** bacterial contamination, metabarcoding, sperm, shotgun metagenomic, sperm quality

## Abstract

Bacterial growth is highly detrimental to sperm quality and functionality. However, during the last few years, using sequencing techniques with a metagenomic approach, it has been possible to deepen the study of bacteria-sperm relationships and describe non-culturable species and synergistic and antagonistic relationships between the different species in mammalian animals. We compile the recent metagenomics studies performed on mammalian semen samples and provide updated evidence to understand the importance of the microbial communities in the results of sperm quality and sperm functionality of males, looking for future perspectives on how these technologies can collaborate in the development of andrological knowledge.

## 1. Introduction

Microorganisms are widely distributed in nature and have colonized almost every environment, including other living organisms. All multicellular organisms harbor microbial communities in and on their bodies, and these microbiomes can significantly influence host biology. Previously, the microbiome was studied from a pathological perspective. However, today it is also studied as a beneficial entity for the host, as it directly influences the health, physiology, development, behavior, and evolution of the host (Rowe et al., 2020).

Microbial communities have co-evolved in animal organisms and are found in almost every body part (Comizzoli and Power, 2019). Most research has focused on different mammals’ oral, skin and gut microbiomes, while relatively little is known about the reproductive microbiome (Rowe et al., 2020). The reproductive microbiome is dynamic, varying both within the genus of the animal, the section of the reproductive tract in which it is established, between species, and within a species over time (Schoenmakers et al., 2019).

Community dynamics are determined by local interactions, immigration from other microbiomes, and the external environment (Rowe et al., 2020). In mammals, exposure to the maternal microbiome determines the offspring’s microbiome, affecting their health later in life (Comizzoli et al., 2021). This is why the microbiome has played, and will continue to play, a fundamental role in the evolution of mammals. They have evolved to endure within their hosts but have provided environments and conditions conducive for the host to evolve (Comizzoli et al., 2021). Also, the microbiome is strongly influenced by environmental factors, and it can be modified based on animal’s environment or living conditions (Comizzoli and Power, 2019).

Microbiomes can influence and evolve for reproductive success, including, for example, in mate selection in the success of offspring. Hence, microbes of the mammary gland, for example, are closely related to the reproductive microbiome (Comizzoli et al., 2021). In fact, Red Queen coevolutionary theory proposes that the microbiomes of semen and vaginal fluids should reach a certain level of homogeneity. Long-term sexual coevolution should favor homogeneity and be beneficial for sexual reproduction, such as sperm survival or fertilization on a physiological/ecological scale (Ma and Taylor, 2020).

Healthy males usually do not contain bacteria in their semen. Nonetheless, preputial diverticulum, skin, and hair contain several microorganisms that can influence when collecting samples as part of fertilization procedures. Also, the collection environment or human intervention can contribute to the contamination of semen samples (Kuster and Althouse, 2016). Therefore, the percentage of bacterial-contaminated semen samples is usually high, which was reported to be up to 66.7% in the case of boars (Schulze et al., 2015). Microbes have recently been shown to significantly affect males’ reproductive function and performance (Alqawasmeh et al., 2022; Poole et al., 2023). Therefore, knowledge of the reproductive microbiome is fundamental to understanding the evolutionary ecology of reproductive strategies and sexual dynamics of host organisms.

**FIGURE 1:**
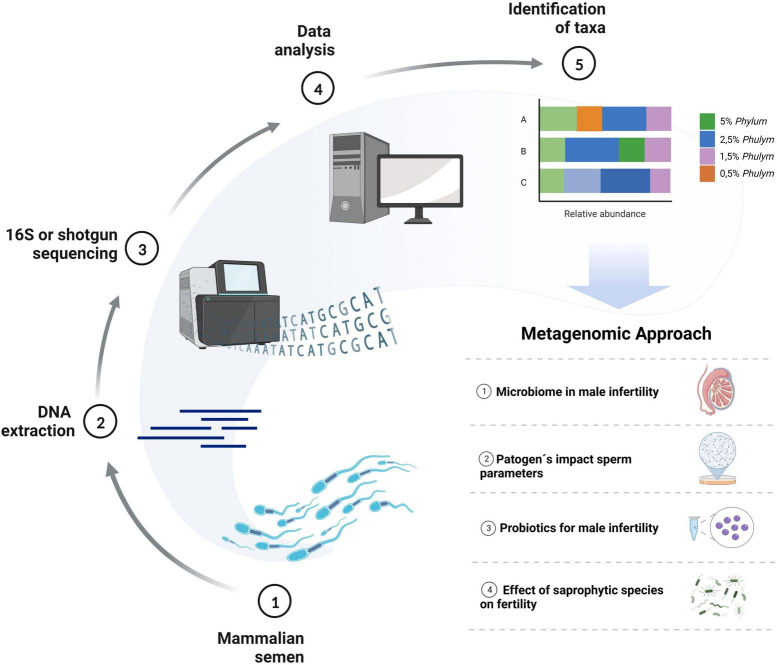
Metagenomics’ approach to future studies in mammalian reproduction. Diagram describing the experimental design and prospects for using metagenomics and sequencing technologies to develop reproductive knowledge. Created with BioRender.com.

In recent years, the work on microbiological diversity in semen samples has been limited to specific bacteria species. However, new sequencing technologies have recently made it possible to study the whole microbial communities in the samples and evaluate their interactions and effect on spermatozoa (Aderaldo et al., 2022). Such studies can have several approaches in semen samples (Figure 1), among them: (a) to study the relationship between testicular and semen microbiome in male infertility; (b) to determine pathogens that negatively impact sperm parameters such as sperm count, motility, morphology, and DNA integrity; (c) to identify probiotics with potential benefits as an alternative therapeutic option for male infertility, such as *Lactobacillus*, recently proposed to have protective effects on semen parameters (Alqawasmeh et al., 2022) and (d) to understand the interactions, protective and deleterious effects of saprophytic species. This review aims to compile the recent metagenomics studies performed on mammalian semen samples and to provide updated evidence to understand the importance of the microbial communities in the results of sperm quality and sperm functionality of males, looking for future perspectives on how these technologies can collaborate in the development of andrological knowledge.

## 2. Metagenomic as revolution microbiome study approach

The traditional dependant-culture technique for the study and identification of microbial isolates is no longer suitable for studies of microbial communities because it may underestimate the presence of some difficult-to-culture bacteria. In this sense, isolation allows the identification of only a small portion (<1%) of a complex community (Almeida and de Martinis, 2019). In addition, phenotypes may vary according to species niches, leading to discordant phenotypic results that limit classical taxonomy. In response to this need, metagenomics brought a solution for culture-independent studies. Metagenomics has defined the study of whole genetic material recovered directly from a sample and through sequencing of marker amplicons –metabarcoding– or whole DNA random fragmentation –shotgun– (Garza and Dutilh, 2015). Recent advances in high-throughput sequencing and the considerable reduction of analysis costs have boosted the development of microbiology and metagenomics (He et al., 2022). Next-generation sequencing (NGS) technologies have made it possible to improve the coverage and depth of studies, allowing the rigorous analysis of complex microbial communities (Almeida and de Martinis, 2019). In amplicon-based metagenomics or metabarcoding, conserved regions of phylogenetic markers (such as 16S or 18S rRNA) are amplified by PCR, sequenced, and assigned to an operational taxonomic unit (OTU) or amplicon sequence variants (ASVs) over the sequenced gene region (Callahan et al., 2017). These OTUs/ASVs can be classified into different taxonomic levels, from phylum to species. In contrast, for shotgun metagenomics, the microbial DNA from the entire sample community is fragmented and sequenced directly using random primers. One of the advantages of shotgun metagenomics is the possibility of obtaining a species-level classification and in-deep study of bacterial genomes/genes on the samples (Ranjan et al., 2016). Besides the identification and abundance study of OTUs/ASVs present in the samples, metagenomics allows for calculating and comparing diversity indices, abundance, and species richness (e.g., Shannon, Simpson, Chao, etc.) (Ma and Li, 2018).

## 3. Metagenomic studies in mammals’ semen

The study of bacteriospermia has had different objectives depending on the mammalian species under study. In the case of humans, the works are focused on avoiding sexually transmitted diseases due to microbial pathogens and the influence of those on sperm quality and possible causes of infertility. In the case of farm animals, the focus is generally on optimizing sperm quality artificial insemination and avoiding the transfer of microorganisms that may affect the female and offspring. The metagenomic studies found to date by this review are detailed below by species.

### 3.1. Humans

**FIGURE 2:**
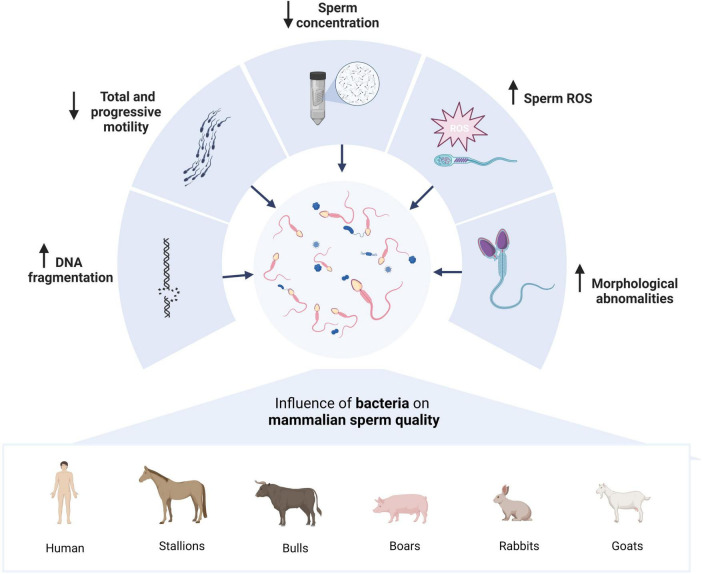
Effects of the relationship between mammalian spermatozoa and the microbiota. Several studies report similar effects on mammalian semen quality parameters such as DNA fragmentation, decreased sperm concentration and progressive and total motility, and increased ROS concentration and morphological abnormalities. Created with BioRender.com.

Infertility affects around 30% of couples worldwide, and treatments can be stressful, intrusive, and expensive (Wagner et al., 2023). Metagenomics is enhancing our knowledge about the composition and role of microbiota in human physiology and pathology. Human semen, just like the female genital tract (vaginal, endometrial, placental), is not sterile and hosts a specific microbiota whose dysbiosis could play a pivotal role in infertility (Venneri et al., 2022). The seminal microbiota consists mainly of bacteria (71.3%), with the largest abundance of *Bacillus, Staphylococcus, Mycobacterium*, and *Streptococcus*, but it also is possible to find Eukaryotes (27.6%) and viruses (1.1%) (Aderaldo et al., 2022). The metagenomic comparative analysis of the seminal microbiota between fertile and infertile men revealed a relative difference in the presence of the *Propionibacteriaceae* family and the *Cutibacterium, Rhodopseudomonas*, and *Oligotropha* genera; also, a negative correlation was detected between the abundance of *Moraxella, Brevundimonas*, and *Flavobacterium* with sperm DNA fragmentation (Garcia-Segura et al., 2022). Recently, a meta-analysis (Farahani et al., 2021) that included fifty-five observational studies through culture, PCR and metagenomic studies, with 51.299 subjects, identified aerobic, facultative anaerobic and strictly anaerobic bacteria, one of them was *Lactobacillus* whose presence was associated with improvements in semen parameters (Yao et al., 2022). This is consistent with that patient with leukocytospermia have a characteristic semen microbiota composition, with a decrease in *Lactobacillus*. In contrast, patients with a normal seminal leukocyte count were categorized as *Lactobacillus*-enriched (Yao et al., 2022). Noticeably, Lactobacillus species have previously been associated with sperm elongation, and Kruger’s strict morphology (this assay provides a useful diagnostic tool in male infertility), which indicates that it may have a significant influence not only on semen morphology but also might be a potential probiotic for semen quality maintenance (Weng et al., 2014; Gachet et al., 2022). Okwelogu et al. (2021) also reported that semen samples colonized by *Lactobacillus jensenii* significantly improve IVF performance. Also, the meta analysis study concludes that Bacteriospermia decreases sperm concentration and progressive motility (PM) and increases DNA fragmentation index; as an example, *Enterococcus faecalis* negatively impact total motility (TM), and *Mycoplasma hominis* reduces concentration and PM and causes an increase in morphological abnormalities (Figure 2; Farahani et al., 2021).

There are many metagenomic studies in which the effect of bacteria on sperm function is evaluated. Some studies have reported ten bacterial genera as testicle sperm-specific, including *Blautia, Clostridium*, and *Prevotella* (Molina et al., 2021). Other studies where a seminal sample was used (Yang et al., 2020; Campisciano et al., 2021), proposed that the testicle harbors its unique low-biomass microbial signature and could be one source of the seminal microbial composition. *Prevotella* was identified in over 90% of testicular samples (Molina et al., 2021); this relative abundance of *Prevotella* increase in semen samples with defective sperm motility and morphology, however overall bacterial content of sperm might not play a major role in male infertility (Baud et al., 2019). Men with fertility problems and categorized as oligospermia tend to have more *Prevotella, Escherichia, Lactobacillus, Shuttleworthia, Serratia, Megasphaera, Gardnerella*, and *Sneathia*; azoospermic men have more *Lactobacillus, Enterococcus, Corynebacterium, Veillonella, Gardnerella, Ureaplasma*, and *Prevotella;* while men with leukocytospermia or pyospermia have a larger abundance of *Gardnerella* and *Prevotella* (Okwelogu et al., 2021). Also, in a study that included 770 men with subfertility, *Enterococcus faecalis* was the most prevalent bacteria in semen (22.0% samples), followed by *Ureaplasma* spp. (16.9% samples), which was related to higher ROS and DNA fragmentation (Ho et al., 2022). Patients that present oligoasthenoteratozoospermia and/or seminal hyperviscosity have augmented at least two times *Pseudomonas, Klebsiella, Aerococcus, Actinobaculum*, and *Neisseria*, which gives a closer link between the presence of these pathogens and infertility (Monteiro et al., 2018). Interestingly, in the same patients, it was observed a lower prevalence of *Lactobacillus* and *Propionibacterium* probiotics (Monteiro et al., 2018). Moreover, semen samples from thirty-one healthy men revealed that HPV-positive semen samples (19.4%) exhibited altered bacterial microbiota composition, including higher abundances of *Moraxellaceae, Streptococcus*, and *Peptostreptococcus* (Tuominen et al., 2021).

These studies provide relevant information evidencing the potential roles of seminal microbiota; nonetheless, it is still necessary to identify which of those microbes are passing by, residents, intruders, or external contamination; the correlation of specific taxa with infertility; and the microbiota dysbiosis effects as a cause of infertility. For example, in a female’s reproductive tract, immune cells can sense the presence of microbes through their pattern recognition receptors; this interaction will activate the immune response and, with it, a series of events that will define the success or failure of fertilization. If seminal microbiota can have such an influence are to be studied.

### 3.2. Stallions

Equine artificial insemination is more often performed with liquid semen than with frozen semen. This is because many factors affect the quality of semen during cold storage, such as the presence of bacteria and the addition of antibiotics (Al-Kass, 2019). Besides, there is a high rate of individual variation between stallions, which makes it challenging to obtain adequate results for the production system (Contreras et al., 2020, 2022). Bacteria colonize semen from stallions during collection and processing, which may affect the inseminated females or negatively affect sperm quality during storage before insemination. To avoid contamination, antibiotics are routinely added to semen extenders to control the growth of these bacteria, but they induce antimicrobial resistance mechanisms daily. Therefore, the metagenomic characteristics of semen samples have been studied, and the relationship that this may have on sperm quality and functionality has been explored (Al-Kass, 2019). A study determines the commensal microbiota of seminal samples from stallions, observing that there are nine main phyla, the most abundant being *Bacteroidetes* (46.50%), *Firmicutes* (29.92%), and *Actinobacteria* (13.58%). At the family level, 69 bacterial families have been described, but only nine are common in all samples from several individuals; *Porphyromonadaceae* (33.18%), *Peptoniphilaceae* (14.09%), *Corynebacteriaceae* (11.32%), and *Prevotellaceae* (9.05%) are the most representative, while the phylum *Firmicutes* has the highest number of families. In addition, high inter-subject variability is confirmed. The study also shows a different trend from other species, such as humans, since *Lactobacillaceae, Staphylococcaceae*, and *Streptococcaceae* only represent 0.00, 0.17, and 0.22% of abundance in stallion sperm (Quiñones-Pérez et al., 2021). Another study determined that there are 83 bacterial genera identified in seminal samples from stallions, varying from 25 to 52 among different individuals. The most abundant are *Porphyromonas* spp., *Corynebacterium* spp., *Peptoniphilus* spp., *Mobiluncus* spp., *Chondromyces* spp., *Suttonella* spp., *Acinetobacter* spp., *and Campylobacter* spp. Also, there was no association between bacterial count and stallion age. At the same time, there was a negative correlation between *Treponema* spp. and *Advenella* spp., because these two microorganisms were never present in the same animal (Al-Kass et al., 2020).

A correlation between seminal microbiota and sperm quality, and even infertility, has been reported in other species. In stallions, a study examined the associations of bacterial presence with five sperm quality parameters: concentration, total sperm count, total and progressive motility, and DNA fragmentation (Figure 2). Only two families appear to correlate with two sperm quality parameters. *Peptoniphilaceae* correlates positively with total sperm motility, while *Clostridiales* (*Incertae Sedis XI*) negatively correlates with progressive motility. This suggests that the seminal microbiome can affect spermatozoa activity in equine spermatozoa. Contrary to initially thought, the correlation is not always negative; saprophytic bacterial flora has a positive activity on some sperm parameters (Quiñones-Pérez et al., 2022). On the other hand, to study the relationship between sperm and mare microbiota, the possibility that contagious equine metritis is caused by males carrying *Taylorella* in apparently agent-free farms was analyzed. Therefore, the comparative microbiota between the carrier and non-carrier stallions of contagious equine metritis was studied, determining that carrier stallions contain a strong predominance of *Corynebacteriaceae* (37.75%) and *Peptoniphilaceae* (28.56%). While in non-carrier males, the most abundant were *Porphyromonadaceae* (20.51%), *Bacteroidaceae* (19.25%), and *Peptoniphilaceae* (18.57%). Therefore, the composition of the seminal microbiome varies when an individual is a carrier of *Taylorella* (Quiñones-Pérez et al., 2020).

### 3.3. Bulls

Until very recently, there were no seminal metagenomics studies in healthy bulls. As a first approach, a study was conducted to identify bacteria in semen from healthy bulls by 16S sequencing, to explore the bacterial communities composition between individual bulls, and to establish whether there was a relationship with fertility. A total of 107 bacterial genera were identified in this study, with negative correlations between *Curvibacter, Rikenellaceae RC9-gut-group*, and *Dyella* spp. compared to *Cutibacterium*, *Ruminococcaceae UCG-005*, *Ruminococcaceae* UCG-010, and *Staphylococcus*. Most bacteria were found to be environmental organisms or species from animal and human mucosa, and two genera, *W5053* and *Lawsonella*, were enriched in low-fertility bulls. Therefore, differences in the seminal microbiota of healthy bulls could be related to fertility (Cojkic et al., 2021).

There is also concern about bacterial contamination of semen as an important factor related to the health status of bulls that can significantly affect artificial insemination. Some important bovine diseases can be transmitted through semen. Therefore, the natural variability of the bacteria between individuals was determined, and the most predominant phyla were *Firmicutes* (31%), *Proteobacteria* (22%), *Fusobacteria* (18%), *Actinobacteria* (13%), and *Bacteroidetes* (12%). In addition, two significant groups of individuals were observed; the first group’s microbiome was based on *Actinobacteria* and *Firmicutes*, while the second group had a high prevalence of *Fusobacteria*. Therefore, there is individual variability between different bulls, so the influence of those microbial patterns on sperm quality must be studied (Medo et al., 2021). The bacterial composition of semen from bulls with satisfactory (S) or unsatisfactory (U) semen quality, represented by poor sperm motility and/or morphology, was recently evaluated to determine whether the diversity and composition of the microbiota may be associated with decreased semen quality. Thirty-two bulls with S spermiograms were matched with 13 U bulls. The most abundant genera in the seminal microbiome were *Bacteroides, Corynebacterium 1, Escherichia, Gemella*, and *S5-A14a*. S bulls had a higher abundance of *Bacteroides, S5-A14a, Trueperella*, *Methanosphaera*, and *Methanobrevibacter*. Comparatively, U bulls had a higher abundance of sequence types belonging to *Veillonellaceae, Campylobacter* and *Methanobacterium*. Also, *Methanobrevibacter* seems to be decisive for maintaining the microbiota, being abundantly present. Opportunistic pathogens such as *Campylobacter* and *Fusobacterium* seem to work in synergy with other microbial community members, but only in the unsatisfactory group. Therefore, the microbiota has a close relationship with sperm quality and exerts a synergistic and/or antagonistic effect depending on the genus (Koziol et al., 2022).

### 3.4. Boars

In the first study on seminal metagenomics, the seminal porcine microbiota before and after diluting different semen production artificial insemination centers was evaluated. It determined the relative abundance of various species and diversity indicators from 7,026,497 OTUs, where the most important families were *Ruminococcaceae* (French studs) and *Pseudomonadaceae* and *Enterobacteriaceae* (American studs). It was observed that there is a direct relationship between production parameters influencing the microbial composition of porcine seminal fluid. Furthermore, the boar stud influenced the composition of this microbiota. Also, one of the variables most associated with this composition was the type of soil on which the animal is housed. Finally, they compared American and French boars, finding different microbiota between them, not associated with the country of origin but rather with the production center (Even et al., 2020). Another study determined the bacterial composition and changes in winter and summer ejaculated boar semen and the underlying mechanism of decreased sperm quality and summer fertility capacity. *Proteobacteria* (57.53%), *Firmicutes* (31.17%), *Bacteroidetes* (4.24%), and *Actinobacteria* (3.41%) are the dominant phyla in boar ejaculated semen, and the dominant genera were *Pseudomonas* (34 41%) and *Lactobacillus* (19.93%). The greater diversity of bacteria in ejaculated winter semen differs from that in summer semen, possibly due to seasonal changes in semen quality and sperm fertilization capacity. A higher abundance of *Lactobacillus* was found in winter samples, positively associated with sperm quality and reproductive performance obtained from sows inseminated with such semen samples. Conversely, the abundance of *Pseudomonas* in summer samples was negatively associated with sperm quality and reproductive potential. Therefore, it has been determined that the microbiota varies according to the seasons in swine, which might be a driving factor for conditions such as “summer infertility” (Zhang et al., 2020).

### 3.5. Rabbits

In the case of rabbits, the effects of sperm microbiota and sperm quality had not been previously described. A recent study evaluated how rabbit male genetics influence sperm microbiota diversity by considering the symbiotic bacteria of four inbred lines of rabbits and their effects on sperm. The symbiotic bacteria of four commercial inbred lines bred in the same facility and their impact on sperm quality and fertility were analyzed. It was determined that the main bacteria belong to *Proteobacteria*, *Firmicutes*, *Fusobacteria*, and *Bacteroidetes* phyla in the sperm microbiota. In addition, at the genus level, the composition of the bacterial community in the sperm microbiota was influenced by host genetics. Also, the number of genera detected is variable among the different lines. They observed that *Enhydrobacter, Ferruginibacter, Myroides Paracoccus, Rheinheimera, Tepidiphilus, Tetradesmus obliquus*, and *Thauera* were only present in inbred lines selected by litter size. Furthermore, the discriminant analysis revealed *Lysinibacillus* and *Flavobacterium* as potential fertility biomarkers. Therefore, sperm microbiota varies between individuals and genetic lines; so, fertilization results differ between different groups (Marco-Jiménez et al., 2020).

### 3.6. Goats

A recent study evaluated the microbial composition of goat semen between reproductive season and anestrus. The five dominant phyla were *Firmicutes, Proteobacteria, Fusobacteria, Actinobacteria*, and *Bacteroidetes* during the breeding season and *Firmicutes, Proteobacteria, Actinobacteria, Bacteroidetes*, and *Cyanobacteria* during the non-breeding season. A decrease in the relative abundance of the genus *Faecalibacterium* and an increase in the genera *Sphingomonas* and *Halomonas* was demonstrated in the ejaculates collected during the reproductive season. The abundance of *Sphingomonas* and *Faecalibacterium* were favorably and unfavorably correlated with sperm quality, respectively. Therefore, there is a variation in the microbiota within the seasons in goats, where the microbiota remains stable for 7 days within a season. Finally, the authors postulate that the genera *Sphingomonas* and *Faecalibacterium* could be possible biomarkers of semen quality in male goats (Mocé et al., 2022).

## 4. Challenges and future perspective of novel metagenomics approaches

One of the crucial challenges in the study of reproductive microbiomes is the limited knowledge we have of these microbial communities. It is generally unclear how reproductive microbiomes are established and maintained (dynamics); moreover there are only few studies regarding the role they may play in host and the functional reproductive processes (Altmäe et al., 2019; Rowe et al., 2020). As described in this review, research on these topics is still at early stage, being more developed in humans than other mammals; it is even more limited in wild species (Comizzoli et al., 2021). Furthermore, due diversity of microorganisms and the conditions affecting the microbial community composition, it has not been possible to determine precisely the native reproductive microbiomes or whether they are more related to transient and random colonization. In this context, another challenge is to develop techniques to allow the discrimination between microorganisms from the seminal core vs. contaminant microorganisms from surrounding environment (Rowe et al., 2020). The control of sample contamination is currently a weak and complex point in most metagenomic studies. In fact, assisted reproduction methodologies are never carried out under sterile conditions (Molina et al., 2021). Therefore, rigorous method needs to be applied to keep the samples clean during the collection process.

In addition, metagenomics design must include a rigorous and strict process that eliminates contamination data from other sources, since the actual microbiome could be present at low proportions compared to contaminant microbes (Molina et al., 2021). It has also been determined in recent times that there is a strong influence of nature and hormone levels on the reproductive microbiome, which in turn affects fertility outcomes, but this constitutes a broad spectrum of possibilities and difficulties for microbiological study (Comizzoli et al., 2021). Another current challenge is the low microbial biomass characteristic of ejaculates and determining the origin of the seminal microbiome (Rowe et al., 2020). It was previously believed that semen was sterile and that the microbiome aggregated as semen moved through the seminiferous ducts until ejaculation; however, recently, in humans, a very small microbial biomass has been confirmed in the testis, which appears to be important in spermatogenesis and cell differentiation (Molina et al., 2021). This represents a challenge in methodologies and data analysis. With such low microbial biomass, systematic control and elimination of possible contamination are crucial to obtain reliable microbiome data on host information and minimizing misinterpretation of results (Molina et al., 2021).

Advances from culture-based methods of bacterial identification to specific sequencing of the 16S rRNA gene [next-generation sequencing (NGS) platforms] in association with the “culturomics” (diversification of culture conditions, combined with identification by matrix-assisted laser desorption/ionization mass spectrometry-MALDI-TOF) have recently begun to increase the cultivable bacterial repertoire of a biological sample (Al-Kass et al., 2020). Furthermore, the revolutionary advances of metagenomics used to understand the complex microbial associations and dynamics in different mammals enlighten the importance of unexplored sperm microbiota and their influence on sperm quality. Importantly, the overall advances of the metagenomic process have been related to improvements in NGS and bioinformatic approaches used for the analysis of the generated sequence read data, providing a platform for discoveries—thus, novel strains that can be further screened for their different functional aspects like potential in antimicrobial agents and therapeutic compounds. Metagenomic NGS (mNGS) workflows sequence as much DNA and/or RNA as possible in a sample, which in practice allows for rapid and accurate screening of microorganisms presents in a sample, strongly impacting clinical and fieldwork (Gaston et al., 2022).

There are multiple bioinformatics approaches to metagenomic data processing, most notably assembly-based (single-sample assembly and multi-sample assembly) and read-based (merged reads and raw data) approaches. However, it remains to be seen how these approaches differ in analyzing the data and how they affect the interpretation of the results. Multisampling provides more helpful information for specific functional traits at a high cost of computational resources and runtime, but no significant differences in the biological data obtained (Zhou et al., 2023). Therefore, the challenge of choosing an appropriate approach is that researchers must decide between multiple factors, such as the scientific question, the amount of usable information, the computational resources available, and the time available for analysis (Zhou et al., 2023).

In addition, the peak-to-trough ratio (Compute PTR or CoPTR) is another useful approach for studying the patterns of sequencing coverage along a bacterial genome. This metric provides a summary of the distribution of sequencing coverage and can be used to assess the quality and consistency of the data. By reflecting the microbial growth rates, the CoPTR can offer valuable insights into the dynamics of microbial communities and the interactions between microbes and their host (Joseph et al., 2022). Additionally, MIDAS2 offers a step forward in comparison to the MIDAS tool, by enabling the analysis of growingly extensive reference genome databases. It also provides the capability of creating personalized databases and utilizing paired-end reads to enhance the accuracy of single-nucleotide variants (SNV) analysis (Zhao et al., 2022). Also, MetaPhlAn 4, has been released as a new method that allows integrating information from both metagenome assemblies and microbial isolate genomes for improved and more comprehensive metagenomic taxonomic profiling, enabling more profound and more comprehensive microbiome biomarker detection (Blanco-Miguez et al., 2023). When reference information is missing, metagenomic data assembly becomes more complicated. To overcome this challenge, several solutions exist, such as genomic binning, *de novo* assembly, reference-based assembly, and hybrid assembly. Among these methods, genomic binning is considered a cutting-edge approach for analyzing mixed culture metagenomic data (Yang et al., 2021). Tools such as BusyBeeWeb assembles contigs and long nanopore-generated reads alike; this web server provides a wide range of supplementary annotations and visualizations, facilitating the analysis of this type of data (Schmartz et al., 2022). Moreover, metagenomic sequencing methods have provided an increasing amount of data on predicted protein sequences; however, only a tiny fraction of all metagenomic sequences collected have been functionally or structurally characterized, so there was a new approach to study, where metagenomic data are used to predict protein structures and discover proteins, allowing, for example, the in-depth study of antibiotic resistance genes (Hou et al., 2022). Besides, metagenomics allows the assembling of eukaryotic cell genomes, as has already been done in marine plankton (Massana and López-Escardó, 2022).

Despite the increase of metagenomics tools and approaches that allow an in-deep study of microbial community composition, function and dynamics, semen microbial studies are still at an initial level. Most studies have been conducted in humans and are still incipient in other mammals. It is well known that seminal parameters are different among species; hence, advances in human semen knowledge only increase the questioning about similar or even more important interactions happening in other species. Then, there is a current challenge to explore the reproductive microbiome in future research by applying more targeted and mechanistic approaches, including shotgun metagenomic sequencing, metabolomics, and *in vitro* experimentation, aiming to develop new molecular markers of fertility such as seminal plasma proteins and metabolites (Wu et al., 2019; Klein et al., 2022).

There are new insights into the utility of reproductive metagenomic studies. As mentioned, there appears to be a low biomass intratesticular microbiome that has implications for modulating spermatogenesis. It is promising and could be a microbiome modulation tool for possible solutions to male infertility problems. On the other hand, pathogen-saprophyte relationships could be explored in more depth, looking for methodologies to modulate competitive saprophytic microbiota and, thus, potentially, the immune barrier and prevent reproductive tract infections or the transfer of pathogenic bacteria through seminal samples. It is also crucial to investigate the relationship between the estrous cycle, hormone levels, and variations within the microbiome to avoid the usual losses in reproductive biotechnologies in production animals, leading to better production results. Finally, the metagenomic study of the reproductive microbiome is and will be a transcendental tool for the conservation of species, as it will allow the study of evolutionary and adaptive mechanisms that could be involved in the lack of reproductive efficiency and potentially, its intervention could collaborate in the preservation of endangered species. Novel metagenomic studies on mammal semen samples could be the key to improving sperm quality, functionality, and fertility based on microbial interactions. An increase in such studies would impact conception success and thereby enhance animal production, while for humans, we can improve the assisted reproductive technologies problems to contribute to infertility.

## Author contributions

MC: conceptualization, literature collection and review, and writing—original draft. KN-M and KL: revision, editing, discussion, and proofreading of the original draft. PB, AZ, FP, and MG: revision and editing of the original draft. LB: supervision, revision, editing, discussion, proofreading of the original draft, and project administration. All authors read and agreed to the published version of the manuscript.
